# Draft genomes of female and male turbot *Scophthalmus maximus*

**DOI:** 10.1038/s41597-020-0426-6

**Published:** 2020-03-12

**Authors:** Xi-wen Xu, Chang-wei Shao, Hao Xu, Qian Zhou, Feng You, Na Wang, Wen-long Li, Ming Li, Song-lin Chen

**Affiliations:** 1 Yellow Sea Fisheries Research Institute, Chinese Academy of Fishery Sciences, Laboratory for Marine Fisheries Science and Food Production Processes, Pilot National Laboratory for Marine Science and Technology (Qingdao), Qingdao, China; 2 Key Lab of Sustainable Development of Marine Fisheries, Ministry of Agriculture, Qingdao, China; 3 College of Fisheries and Life Science, Shanghai Ocean University, Shanghai, China; 4 Institute of Oceanology, Chinese Academy of Sciences, Qingdao, China

**Keywords:** Genome, Differentiation, DNA sequencing, Ichthyology

## Abstract

Turbot (*Scophthalmus maximus*) is a commercially important flatfish species in aquaculture. It has a drastic sexual dimorphism, with females growing faster than males. In the present study, we sequenced and *de novo* assembled female and male turbot genomes. The assembled female genome was 568 Mb (scaffold N50, 6.2 Mb, BUSCO 97.4%), and the male genome was 584 Mb (scaffold N50, 5.9 Mb, BUSCO 96.6%). Using two genetic maps, we anchored female scaffolds representing 535 Mb onto 22 chromosomes. Annotation of the female anchored genome identified 87.8 Mb transposon elements and 20,134 genes. We identified 17,936 gene families, of which 369 gene families were flatfish specific. Phylogenetic analysis showed that the turbot, Japanese flounder and Chinese tongue sole form a clade that diverged from other teleosts approximately 78 Mya. This report of female and male turbot draft genomes and annotated genes provides a new resource for identifying sex determination genes, elucidating the evolution of adaptive traits in flatfish and developing genetic techniques to increase the sustainability of turbot aquaculture.

## Abstract (summary)

**Table:** 

Measurement(s)	DNA • genome • sequence_assembly • sequence feature annotation
Technology Type(s)	DNA sequencing • sequence assembly process • sequence annotation
Factor Type(s)	sex
Sample Characteristic - Organism	Scophthalmus maximus

Machine-accessible metadata file describing the reported data: 10.6084/m9.figshare.11891598

## Background & Summary

Turbot (*Scophthalmus maximus*) is an economically important flatfish with both eyes on the upper side of the body, and it is commonly found along the Atlantic coast of Europe. Aquaculture of turbot was initiated in Scotland in the 1970s and subsequently expanded into other European countries by the early 1980s^1^. In the 1990s, turbot was introduced to China where its farming has since developed rapidly. China is currently the largest producer of turbot in the world^2^. Turbot growth is sexually dimorphic, with females eventually attaining sizes up to 50% larger than those of males^3^. An all-female stock can potentially increase the production value of turbot aquaculture. The sex determination system of turbot follows the ZW/ZZ model, and this system can be affected by environmental factors^4^. Therefore, understanding the genomic architecture of female and male turbot may enable screening for sex determination loci, improve understanding of the interactions between genetic and environmental factors in sex determination, and lead to the acquisition of genomic resources for molecular breeding. Four sex-related QTLs, located on four different linkage groups, have been found in turbot^5^. Though the turbot genome has been assembled, the sex-determining mechanism of turbot remains unclear^6^.

In this study, we sequenced, assembled and annotated the female and male turbot genomes, and conducted a phylogenetic analysis using the genome sequences of eight other closely related species. A 568 Mb female genome sequence and 584 Mb male genome sequence were assembled. The draft turbot genomes represent a valuable resource for isolating the sex determination genes, increasing our understanding of flatfish development and improving the molecular breeding techniques for turbot.

## Methods

### Turbot samples and genome sequencing

**Fig. 1:**
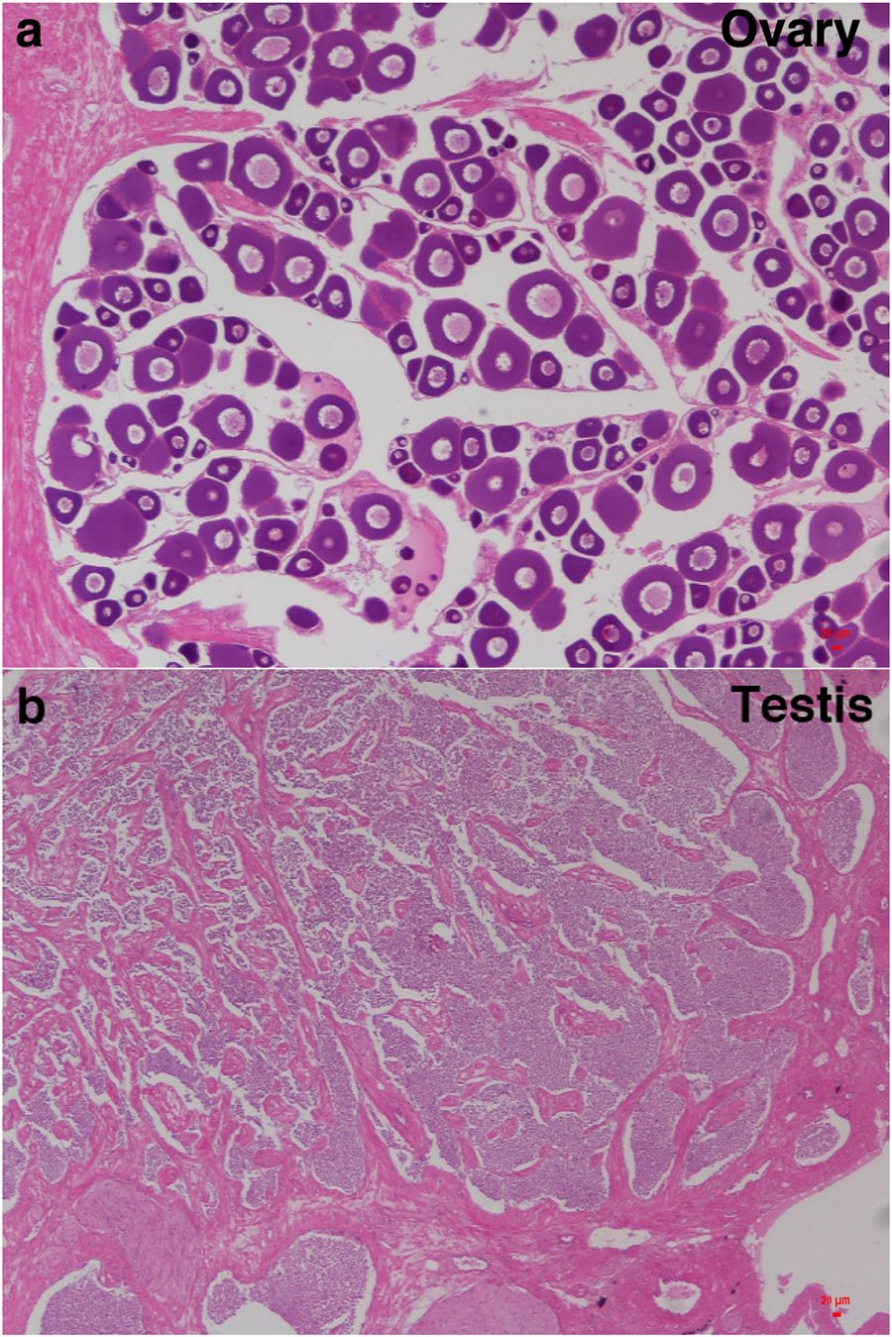
Paraffin sectioning and HE staining of gonadal tissues of the female and male turbot. (**a**) Section of the ovary. (**b**) Section of the testis.

One female (ZW) and one male (ZZ) adult turbot were selected for whole genome shotgun sequencing and were temporarily maintained at 16 °C in laboratory facilities. Subsequently, the physiological sex of each turbot was determined by paraffin sectioning and HE staining of its gonadal tissues (Fig. 1). Blood samples were collected from the subjects using sterile syringes that contained anticoagulant solution (0.5 M EDTA, pH 8.0). Blood samples were stored at 4 °C. High-quality genomic DNA was extracted using Puregene Tissue Core Kit A (Qiagen, USA) for constructing DNA libraries (2 k~40 Kb). We constructed three paired-end (PE) libraries (170 bp, 500 bp and 800 bp) and five mate-paired (MP) libraries (2 kb, 5 kb, 10 kb, 20 kb and 40 kb) for female turbot using TruSeq PE Cluster Kit v3-cBot-HS (Illumina, USA) and Nextera Mate Pair Library Prep Kit (Illumina, USA). The samples were sequenced using the Illumina HiSeq. 2000 platform. We constructed six libraries (PE libraries 170 bp, 500 bp and 800 bp; MP libraries, 2 kb, 5 kb and 10 kb) for male turbot using HiSeq. 3000/4000 PE Cluster Kit and Nextera Mate Pair Library Prep Kit (Illumina, USA), the samples of which were sequenced using the Illumina HiSeq. 4000 platform. In total, we generated 99.5 Gb and 196.4 Gb of raw data for the female and male turbot, respectively. Before genome assembly, we filtered artificial and low-quality reads, resulting in 89.3 Gb and 174.6 Gb of clean data for the female and male fish, respectively (Table 1).

**Table 1:** Summary of sequencing data.

Libraries	Female turbot		Male turbot	
	Total raw data (Gb)	Total clean data (Gb)	Total raw data (Gb)	Total clean data (Gb)
170 bp	20.33	19.75	/	/
230 bp	/	/	65.93	59.85
500 bp	11.02	9.95	49.37	47.58
800 bp	8.91	7.42	19.72	17.9
2 kb	31.08	28.81	16.84	13.38
5 kb	8.39	7.44	22.55	17.81
10 kb	13.01	11.25	21.99	18.08
20 kb	1.86	1.79	/	/
40 kb	4.9	2.88	/	/
Total	99.5	89.29	196.4	174.6

In general, genome size (G) can be calculated following the formula G = K_num/K_depth, where K_num is the total number of k-mer and K_depth is the expected coverage depth of k-mer^7^. We used the 31.3 Gb of female sequence data and the 59.8 Gb of male sequence data to estimate the genome sizes of turbot. The parameters used for the female were: K, 17; K_num, 26,302,164,550; and K_depth, 47. For the male, the parameters were: K, 17; K_num, 53,446,209,674; and K_depth, 95. We estimated the genome sizes to be 559 Mb and 562 Mb for the female and male, respectively. Based on these estimated genome sizes, the high-quality data we obtained covered 159X and 310X the haploid genome of female and male turbot, respectively.

### Genome assembly and anchoring of the pseudo-chromosomes

The turbot genomes were *de novo* assembled using SOAP*denovo*2 (v2.04)^8^ with a parameter of “-K 29”. SOAP*denovo*2 employs the *de Bruijn* graph algorithm to simplify assembly and reduce computational complexity. The gaps were filled using GapCloser (v1.12)^8^ with default parameters. Using this method, we assembled a genome spanning a contig length of 542 Mb, with a contig N50 of 12.16 kb, and a 568 Mb scaffold length, with a scaffold N50 of 6.2 Mb, for the female. The male genome had a contig length of 553 Mb with a contig N50 of 16.52 kb and a scaffold length of 584 Mb with a scaffold N50 of 5.9 Mb (Table 2). The sizes of the draft assemblies in our study are a few Mb larger than that of a previous turbot draft assembly of unknown sex^6^.

**Table 2:** Turbot genome assembly statistics.

Genome assembly	Female turbot	Male turbot
Contig N50 Size (kb)	12.16	16.52
Contig No. (>1 Kp)	73,671	57,539
Longest Contig (kb)	197.81	132.66
Total Contig Length (Mb)	541.51	553.24
Scaffold N50 Size (Mb)	6.17	5.93
Scaffold No. (>1 Kp)	6,292	1,064
Longest Scaffold (Mb)	19.88	19.47
Total Scaffold Length (Mb)	568.45	584.74
GC Content (%)	43.42	43.70

To construct pseudo-chromosomes of the turbot genome, we anchored the scaffolds of the female genome onto linkage groups on two genetic maps: one containing 514 SSRs and the other containing 6,647 SNPs^5,9^. We mapped SSR and SNP markers to the scaffolds using e-PCR and BLASTN (e-value ≤ 1e-5), and we linked the scaffolds that had SSRs and SNPs consistent with those on the maps onto the chromosomal regions, with strings of ‘N’s representing the gaps between adjacent scaffolds. The scaffolds with markers located on different chromosomes were filtered out. In total, 420 scaffolds with 535 Mb lengths were anchored onto 22 chromosomes, and 94% of the scaffolds were used.

### Genome annotation

Transposable elements (TEs) are abundant in vertebrate genomes and contribute to genome evolution^10^. We identified TEs in the female turbot genome using both homology-based and *de novo* prediction approaches. In the homology-based approach, we identified known TEs by searching for regions that match the RepBase TE library (v16.10)^11^ using RepeatMasker (v3.3.0)^12^ and RepeatProteinMask (v3.2.2). In addition, we constructed a *de novo* repeat library of the turbot genome using repeatScout (v1.0.5)^13^. Furthermore, we used the *de novo* library as a reference and used RepeatMasker to further identify TEs. In total, we identified 87.8 Mb of TEs, accounting for 15.44% of the genome, which represents a higher proportion of the genome than do the TEs of other flatfish genomes (56.2 Mb in Japanese flounder and 20.3 Mb in Chinese tongue sole)^14,15^. Among the different types of TEs, DNA transposons were the most abundant (5.77%, 32.8 Mb) (Table 3).

**Table 3:** Predicted levels of differentgenomic repeat elements.

	RepBase TEs		TE Proteins		*De novo*		Combined TEs	
	Length (bp)	% in Genome	Length (bp)	% in Genome	Length (bp)	% in Genome	Length (bp)	% in Genome
DNA	20,826,307	3.66	1,913,179	0.34	16,586,479	2.92	32,827,840	5.77
LINE	8,911,117	1.57	5,680,889	1.00	7,515,445	1.32	13,233,157	2.33
LTR	8,577,908	1.51	2,067,745	0.36	1,931,900	0.34	10,207,971	1.80
SINE	2,054,064	0.36	0	0.00	2,462,561	0.43	2,749,822	0.48
Other	7,610	0.00	0	0.00	5,880,197	1.03	5,887,807	1.04
Unknown	0	0.00	0	0.00	33,943,746	5.97	33,943,746	5.97
Total	36,237,231	6.37	9,656,599	1.70	67,224,071	11.82	87,802,760	15.44

Note: Repbase TEs, the results of RepeatMasker based on Repbase; TE proteins, the results of RepeatProteinMask based on Repbase; *De novo*, the results of RepeatMasker by using the library predicted through *De novo*; Combined, all the results combined.

**Fig. 2:**
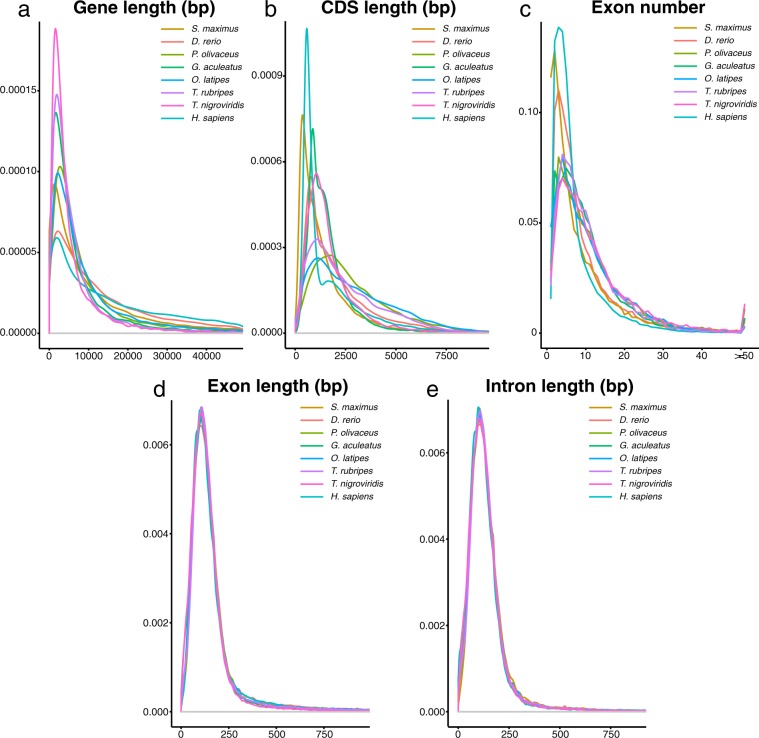
Comparisons of gene parameters among *Scophthalmus maximus*, *Danio rerio*, *Paralichthys olivaceus*, *Gasterosteus aculeatus*, *Oryzias latipes*, *Takifugu rubripes*, *Tetraodon nigroviridis* and *Homo sapiens* genomes. (**a**) Gene length distributions of the species. (**b**) CDS length distributions of the species. (**c**) Exon number distributions of the species. (**d**) Exon length distributions of the species. (**e**) Intron length distributions of the species. Y-axis of (**a**,**b**,**d**,**e**) stand for density, while Y-axis of (**c**) stands for ratio of genes.

We also used homology-based and *de novo* approaches to predict genes in the female genome assembly. For the homology-based prediction, *Danio rerio*, *Gasterosteus aculeatus*, *Oryzias latipes*, *Takifugu rubripes*, *Tetraodon nigroviridis*, *Cynoglossus semilaevis*, *Paralichthys olivaceus* and *Homo sapiens* proteins were downloaded from Ensembl (release 60) and NCBI, and we mapped the protein sequences onto the turbot genome using tblastN (e-value ≤ 1e-5). Homologous genome sequences were aligned against matching proteins using Genewise (v2.4.0)^16^ to define genes. We identified 11,245 to 20,057 homologous genes using the eight species reference. For *de novo* prediction, Augustus (v2.5.5)^17^ and Genscan (v1.0)^18^ were employed to analyze the repeat masked genome, which predicted 24,402 and 28,024 genes, respectively. Additionally, RNA-seq data were mapped to the genome to support 17,688 genes. To combine the results from the various analyses, we used Glean^19^ to obtain a primary non-redundant gene set of 20,134 genes with a mean gene length of 10,322 bp and an average CDS length of 1,605 bp (Table 4). The average exons number per gene was 9.63, and the average length per gene was 166 bp (Fig. 2). For the predicted gene set, we annotated motifs and domains by using InterProScan (v5.16)^20^ against publicly available databases including Pfam, ProDom, SMART, PRINTS, SUPERFAMILY and PROSITE^21^. We identified 18,434 genes containing conserved functional motifs in the predicted protein sequence. We also obtained Gene Ontology (GO) information for the predicted genes and found that 15,837 genes had GO annotations. We mapped the protein sequences from the turbot genome to the Kyoto Encyclopedia of Genes and Genomes (KEGG) pathway maps (KEGG database, release 58.0) using BLASTP (e-value ≤ 1e-5)., which assigned KEGG pathways to 9,930 genes. We also searched the SwissProt and Trembl databases (UniProt, release 2011_06) using BLASTP (e-value ≤ 1e-5), which resulted in a total of 14,806 and 18,441 assigned proteins, respectively. Only 353 genes (1.75%) were not supported by the protein databases.

**Table 4:** Summary of predicted protein-coding genes in the female turbot genome.

Gene set		Number	Average gene length (bp)	Average CDS length (bp)	Average exon per gene	Average exon length (bp)	Average intron length (bp)
*De novo*	Augustus	27,283	11,982	1,373	7.84	175.09	1,551
	Genscan	26,365	15,475	1,579	9.5	166.23	1,635
Homolog	*G. aculeatus*	19,789	9,844	1,540	9.51	161.89	976
	*D. rerio*	17,209	10,649	1,579	9.65	163.73	1,049
	*O. latipes*	20,057	8,619	1,425	8.7	163.86	935
	*H. sapiens*	11,245	14,191	1,710	11.19	152.82	1,225
	*T. rubripes*	18,185	10,426	1,601	9.84	162.71	998
	*T. nigroviridis*	17,043	8,835	1,446	8.99	160.85	925
	*C. semilaevis*	19,749	9,928	1,533	9.14	167.61	1,031
	*P. olivaceus*	20,028	11,190	1,693	9.76	173.5	1,085
RNA-seq	*S. maximus*	17,668	8,050	1,826	8.52	214.3	868
Final set		20,134	10,322	1,605	9.63	166.63	1,010

Note: Gene length includes the lengths of the exon and intron regions but not the lengths of the UTRs. The accession numbers of the RNA-seq data in this study are SRR4853423 and SRR346085.

### Gene family clustering

**Fig. 3:**
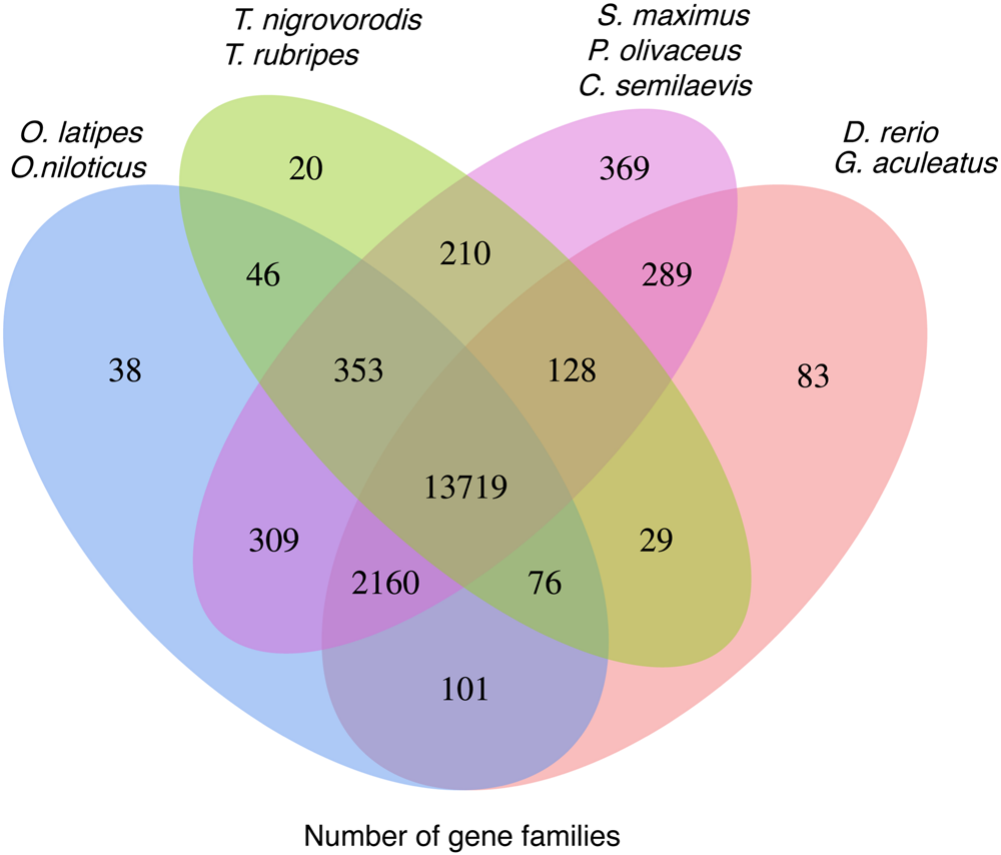
Venn diagram of the numbers of unique and shared gene families among nine sequenced teleost species.

A gene family is a group of genes with similar structures, general with similar functions^22^. We clustered the genes into gene families of turbot and *D. rerio*, *G. aculeatus*, *O. latipes*, *T. rubripes*, *T. nigroviridis*, *C*. *semilaevis*, *P. olivaceus* and *O. niloticus* using OrthoFinder2 (v2.2.7)^23^. A total of 20,134 turbot genes were clustered into 14,440 gene families with an average of 1.39 genes per gene family. We identified 369 putative specific gene families among the three flatfish species included in the analysis. These lineage-specific gene families may have contributed to the evolution of flatfish (Fig. 3).

### Phylogenetic construction and divergence time estimation

For phylogenetic analysis, 3,512 single-copy gene families were defined as orthologous genes by OrthoFinder2 (v2.2.7)^23^. We used MAFFT (v7.427)^24^ for multiple sequence alignment and used trimAL (v1.2)^25^ for automated alignment trimming. Subsequently, we used IQ-TREE^26^ (-m MFP) to reconstruct the phylogenetic tree.

We used the BRMC approach to estimate species divergence times using MCMCTree through the Phylogenetic Analysis by Maximum Likelihood (PAML) package (v4.5)^27^. The MCMC process of the PAML MCMCTree program was run to sample 200,000 times, with sample frequency set to 2 and a burn-in of 20,000 iterations. Other parameters were set at their default values. The calibration times for the *T. rubripes-T. nigroviridis* divergence and *D. rerio*-*O. latipes*, *G. aculeatus*, *T. rubripes*, *T. nigroviridis* (min 149.85 Mya; max 165.2 Mya) were derived from previous research^28^.

**Fig. 4:**
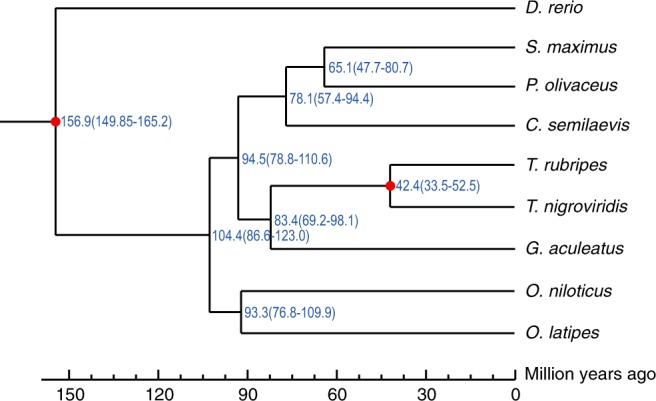
Evolution of orthologous gene families and their estimated divergence times in nine teleost species. The blue numbers on the nodes are the divergence times in million years ago (Mya). The red circles indicated the calibration time.

Our analysis suggests that turbot, Japanese flounder and Chinese tongue sole, all of which belong to Pleuronectiformes, form a monophyletic clade. Our phylogenetic analysis suggests that the turbot and Japanese flounder likely shared a common ancestor approximately 65 million years ago (Mya) and that this ancestor diverged from the Chinese tongue sole approximately 78 Mya (Fig. 4). These findings are consistent with conclusions from previous evolutionary studies^14,29^.

## Data Records

The genomic sequence data have been deposited to NCBI *Sequence Read Archive* (SRA) with the accession number SRP197491^30^. The female genome^31^ and male genome^32^ assemblies are available at NCBI *GenBank*. The whole genome shotgun (WGS) project has the project accession VEVO00000000. This version of the project (01) has the accession number VEVO01000000, and consists of sequences VEVO01000001-VEVO01028256^33^. The list of gene families generated in this work, the annotation gff files of the female genome and the repeat annotations, the alignment file used for constructing the phylogenetic tree and the tree output are available at *Figshare*^34^.

## Technical Validation

### Genome assembly quality assessment

To assess the qualities of the male and female genome assemblies. we first used BWA^35^ with the default parameters to map the PE libraries reads used for assembly back to the corresponding genome, and we used the SAMtools flagstat function (SAMtools v1.9)^36^ to count basic statistics. For the female genome, 99.6% of the PE library reads could be mapped back to the female assembled genome and 96.38% of the mapped reads could be mapped in proper pairs. For the male genome, the re-mapped reads and the reads mapped in proper pairs were 99.75% and 93.23%, respectively. We also calculated the coverage depth of each base pair with the SAMtools depth function (SAMtools v1.9) and found that the coverage depth was greater than 5 for more than 99.11% of male assembly sequences and for more than 96.76% of the female assembly sequences with the exception for the gap areas. We then used Benchmarking Universal Single-Copy Orthologs (BUSCO, v3.0.2)^37^ to assess the assembled genome sequences. We used BUSCO with 4,854 single-copy orthologs from actinopterygii_odb9 to assess the completeness of the female and male turbot genome sequences. For the female genome, 4,427 (96.6%) complete Actinopterygii BUSCOs were present in the female turbot genome, including 4,319 (94.2%) single-copy Actinopterygii BUSCOs and 108 (2.4%) duplicated Actinopterygii BUSCOs. Seventy-two (1.6%) fragmented Actinopterygii BUSCOs were present, possibly due to incomplete assembly, and only 85 (1.8%) Actinopterygii BUSCOs were considered missing in the female genome assembly. For the male genome, 4,467 (97.4%) complete Actinopterygii BUSCOs were present in the male genome, including 94.3% single-copy and 3.1% duplicated Actinopterygii BUSCOs. The fragmented and missing Actinopterygii BUSCOs in male genome represented 1.1% and 1.5%, respectively, of the genome.

**Fig. 5:**
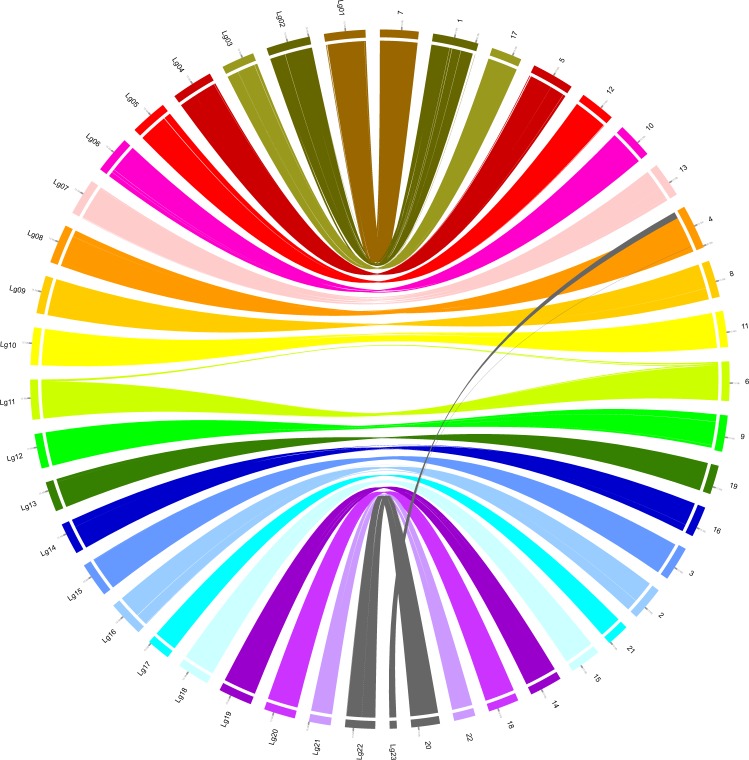
Circos graph of whole-genome synteny analysis for female genome and the reference genome of turbot.

To further validate the technical quality of the new male and female genome assemblies, we used nucmer^38^ to compare our new male and female genome assemblies with the current reference genome assembly (GCA_003186165.1), then used dnadiff^39^ to wrap the comparison results (Table 5). Moreover, LASTZ^40^ with optimized parameters (–hspthresh = 4500 –gap = 600,150 –ydrop = 15000 –notransition) and Circos graph were used to make a correspondence analysis between 23 linkage groups and 22 chromosomes in reference genome. Consequently, 21 linkage groups have one-to-one corresponding chromosomes in reference genome, while Lg08 and Lg23 are both corresponding to chromosome 4 (Fig. 5). The above results indicated that the assembled genome sequences and the gene region assembly are acceptable.

**Table 5:** The comparison between the new male and female genome assemblies and the reference genome assembly of turbot.

	Reference genome	Female genome	Male genome
Total Bases	524,979,463	568,483,288	587,187,767
Aligned Bases	520,165,145 (99.08%)	552,306,146 (97.15%)	562,821,085 (95.85%)
Unaligned Bases	4,814,318 (0.92%)	16,177,142 (2.85%)	24,366,682 (4.15%)

## Data Availability

The data analysis methods, software and associated parameters used in this study are described in the Methods section. Default parameters were applied if no parameter was described. No custom scripts were generated in this work.
